# What do the teachers want? A targeted needs assessment survey for prospective didactic training of psychiatry medical educators

**DOI:** 10.3205/zma001673

**Published:** 2024-04-15

**Authors:** Franziska Baessler, Ali Zafar, Katja Koelkebeck, Thomas Frodl, Jörg Signerski-Krieger, Severin Pinilla, Gottfried M. Barth, Deborah Jannowitz, Sven Speerforck, Daniela Roesch-Ely, Ina Kluge, Miriam Aust, Janine Utz, Gian-Marco Kersten, Philipp Spitzer

**Affiliations:** 1 Heidelberg University Hospital, Department of General, Internal and Psychosomatic Medicine, Center for Psychosocial Medicine, Heidelberg, Germany; 2 Heidelberg Academy of Sciences and Humanities, Heidelberg, Germany; 3 University Duisburg-Essen, Faculty of Medicine, LVR-University Hospital Essen, Department of Psychiatry and Psychotherapy, Essen, Germany; 4 University Duisburg-Essen, Center for Translational Neuro- & Behavioral Sciences (C-TNBS), Essen, Germany; 5 University Hospital Aachen, Department of Psychiatry, Psychotherapy and Psychosomatics, Aachen, Germany; 6 University Medical Center Göttingen, Center for Psychosocial Medicine, Göttingen, Germany; 7 University Hospital of Old Age Psychiatry and Psychotherapy, Bern, Switzerland; 8 Marion von Tessin-Memory Zentrum, Munich, Germany; 9 University Hospital of Tübingen, Department Child and Adolescent Psychiatry, Psychosomatics and Psychotherapy, Tübingen, Germany; 10 Helios Hanse Hospital Stralsund, Clinic for Psychiatry and Psychotherapy, Stralsund, Germany; 11 University of Leipzig, Medical Faculty, Department of Psychiatry, Leipzig, Germany; 12 University of Heidelberg, Department of General Psychiatry, Center for Psychosocial Medicine, Heidelberg, Germany; 13 Marburg University, Department of Psychiatry and Psychotherapy, Marburg, Germany; 14 University of Münster, University Hospital Münster, Department of Psychiatry, Münster, Germany; 15 Friedrich-Alexander University Erlangen-Nuremberg, Erlangen University Hospital, Department of Psychiatry and Psychotherapy, Erlangen, Germany

**Keywords:** medical education, didactics, medical teaching, medical curriculum, train the trainers, psychiatry, psychiatric education

## Abstract

**Objectives:**

Physicians and psychologists at psychiatric university hospitals are assigned teaching tasks from the first day of work without necessarily having the prerequisite training in teaching methods. This exploratory survey provides a needs-based analysis for the prospective didactic training of physicians and psychologists at psychiatric hospitals in Germany, Austria and Switzerland.

**Methods:**

An online questionnaire was distributed at medical schools via email in German-speaking countries in Europe. All physicians involved in teaching medical students at psychiatry faculties were eligible to participate in the survey. Participants were further requested to recruit eligible participants (snowball sampling). Responses were analyzed descriptively, and differences between groups were calculated using nonparametric Mann-Whitney U tests (p<.05).

**Results:**

Overall, 97 respondents (male=55, female=42; mean age= 40.6) from 19 medical schools completed the survey. The respondents consisted of 43 residents, 39 specialists, 6 chief physicians and 9 psychologists. Of the respondents, 97.6% rated didactic competence as either highly relevant or rather relevant for teaching medical students. The highest overall interest was shown for bedside teaching (mode=4; IQR: 2-4) and error culture (mode=3; IQR: 2-4). Respondents expressed the highest training needs for topics regarding presentation and communication (mode=3; IQR: 2-3). Resident physicians were significantly more interested in bedside teaching (U=362.0, p=0.004) and roleplay (U=425.0; p=0.036) than specialist physicians, who were more interested in examination didactics (U=415.0; p=0.022). Chief physicians displayed significantly deeper interest in group dynamics (U=51; p=0.023) than specialist physicians. In-person training was preferred by a majority of respondents, and 27.4% preferred online/web-based training.

**Conclusions:**

The majority of physicians and psychologists at psychiatric university hospitals considered professional development for faculty to be helpful for teaching medical students. Bedside teaching and error culture management were the most desired teaching topics for training medical teachers. Tailored educational interventions are recommended, with target-oriented priorities for different hierarchical levels.

## Zusammenfassung

**Zielsetzung:**

Ärzt*innen und Psycholog*innen an psychiatrischen Universitätskliniken werden vom ersten Arbeitstag an mit Lehraufgaben konfrontiert, ohne jedoch notwendigerweise die hierzu erforderliche didaktische Ausbildung erhalten zu haben. Diese Pilotstudie liefert eine bedarfsorientierte Untersuchung für die zukünftige didaktische Ausbildung an psychiatrischen Kliniken tätigen Ärzt*nnen und Psycholog*nnen in Deutschland, Österreich und der Schweiz.

**Methodik:**

Eine Onlineumfrage wurde per E-Mail an die Medizinischen Fakultäten in deutschsprachigen, europäischen Ländern verteilt. Alle an der studentischen Lehre beteiligten Ärzt*innen an psychiatrischen Kliniken waren teilnahmeberechtigt. Die Proband*innen wurden außerdem gebeten, qualifizierte Teilnehmer*innen zu rekrutieren (Schneeballverfahren). Die Rückmeldungen wurden deskriptiv ausgewertet, Gruppenunterschiede wurden mit nonparametrischen Mann-Whitney-U-Tests berechnet (p<.05)

**Ergebnisse:**

97 Teilnehmende (männlich=55, weiblich=42; mittleres Alter=40,6 Jahre) von 19 Medizinischen Fakultäten schlossen die Umfrage ab. Diese setzten sich aus 43 Assistenzärzt*innen (AÄ), 39 Fachärzt*innen (FÄ), 6 Chefärzt*innen (CÄ) sowie 9 Psycholog*innen zusammen. 97,6% der Proband*innen empfanden didaktische Kompetenz als relevant, oder sehr relevant für die studentische Lehre. Das größte Interesse bestand an Bedside Teaching (Modus=4; IQA: 2-4) und Fehlerkultur (Modus=3; IQA: 2-4). Der größte Ausbildungsbedarf zeigte sich bei Themen um Präsentation und Kommunikation (Modus=3; IQA: 2-3). Assistenzärzt*innen waren signifikant interessierter an Bedside Teaching (U=362; p=0,004) und Rollenspielen (U=425; p=0,036) als Fachärzt*innen, welche Prüfungsdidaktik als relevanter einstuften (U=415; p=0,022). Chefärzt*innen waren signifikant mehr an Gruppendynamik (U=51; p=0,023) interessiert als Fachärzt*innen. Die Mehrheit der Teilnehmer*innen bevorzugte Präsenzangebote, 27,4% Onlineangebote.

**Schlussfolgerungen:**

Der Großteil der Ärzt*innen und Psycholog*innen an psychiatrischen Universitätsklinika bewerteten medizindidaktische Fortbildungen als hilfreich für die studentische Lehre. Die meistgewünschten Themen waren hierbei Bedside Teaching und Fehlerkultur. Empfohlen werden zielorientierte, auf die unterschiedlichen Hierarchieebenen speziell zugeschnittene Seminare.

## Introduction

All medical school graduates are expected to teach as residents and eventually as faculty members at teaching hospitals. Since medical schools do not provide formal educator training to medical students, most physicians do not gain the didactic skills needed for teaching [1], [2], [3], [4], [5]. While all physicians should gain basic teaching and learning skills during their education and training [6], [7], [8], the exact requirements may vary according to the needs of the role and responsibilities, for instance, training in interpersonal skills specifically for education in psychiatry [9]. According to the Canadian physician competency framework (CanMEDS) [10], competent physicians must effectively integrate their abilities under seven different roles as medical experts, communicators, collaborators, leaders, health advocates, scholars and professionals. A recent review of healthcare educator competencies identified six core domains of competence related to teaching and facilitating learning: designing and planning learning; assessment of learning; educational research and scholarship; educational leadership and management; and educational environment, quality, and safety [11]. While physicians, especially those involved in teaching students, must develop wide-ranging skills, attitudes and practices to become competent teachers, curriculum planners should also ensure that the teaching methods employed at medical schools improve the skills of future physicians.

In many countries, knowledge of effective teaching techniques now comprises the learning goals for graduate physicians, such as those described under the German national guidelines for medical education (NKLM – Nationaler Kompetenzbasierten Lernzielkatalog Medizin) [12], the UK’s General Medical Council guidelines [13] or the Principal Relevant Objectives and Framework for Integrative Learning and Education in Switzerland (PROFILES) [14]. Several frameworks have been developed to set out the knowledge, skills and characteristics required of medical teachers in various roles and environments [15], [16]. Similarly, medical schools have also realized the importance of teacher training and introduced didactics courses for teaching staff [1]. A majority of German medical schools now offer educational and instructional skills training for their teaching staff [17]. Studies suggest, however, that exposure to teaching principles, skills and techniques should be done in a sequential manner during physician training, starting in medical school and continuing through postgraduate education and into practice [1], [18].

Teacher training workshops are relatively simple and comprehensive means for bridging knowledge gaps and enhancing the instructional skills of faculty members [19]. Medical education workshops can not only offer an opportunity for senior physicians to cultivate their educational theoretical knowledge and teaching skills but can also contribute positively to the learning experiences of medical students [20], [21]. Several studies have shown their effectiveness in improving the didactic skills of medical teachers from different teaching backgrounds and specialties [20], [22], [23], [24], [25]. However, there has not been a needs-based analysis of what physicians and psychologists in psychiatric hospitals at different stages of their careers want to learn to improve their didactic skills.

This exploratory survey aims to provide an overview of the needs for the prospective didactic training of physicians in Germany, Austria and Switzerland. The findings may serve as groundwork for designing and implementing a core, purpose-designed training workshop for physicians in German-speaking countries (see attachment 1 in German).

## Methods

A cross-sectional online survey was conducted at medical schools in Germany, Austria and Switzerland. An introductory text containing information about the study objectives, confidentiality and anonymity of data, and voluntary participation was included within the questionnaire. All participants provided informed consent at the beginning of the questionnaire, which was self-administered anonymously. All participants agreed to the anonymous scientific use of the data obtained. The survey was conducted according to the principles of good scientific research described by the Declaration of Helsinki [26]. The ethics committee of Friedrich-Alexander-University Erlangen-Nuremberg stated that no approval was required for studies employing anonymous data collection methods under Section 15 (Research) of the Professional Code of Conduct for Physicians in Bavaria, Germany [27].

### Participants

Chief physicians and/or teaching coordinators of 54 medical schools (43 in Germany, seven in Switzerland and four in Austria) were contacted via email and asked to share the link to the survey among their teaching staff (snowball sampling) [28], [29]. Physicians and psychologists involved in teaching medical students in the psychiatry department of a medical school were eligible to participate in the survey. Primary inclusion criteria were teaching psychiatry at a medical school and consent to participate (age>18 years). Since the questionnaire was in German, proficiency in the German language was also a prerequisite for participation.

### Questionnaire

An interdisciplinary research group comprising senior physicians, psychiatrists and medical educators designed a descriptive online questionnaire for the study after group Delphi method discussions [30] using Miller’s pyramid for the assessment of clinical competency [31]. In consultation with experts in the field of didactics in psychiatry, we used the nominal group technique for brainstorming and prioritizing ideas to formulate questions. The questions were derived from different training modules, didactic recommendations and research findings on curriculum planning. After two rounds of discussions, comments and adaptions, the questions were finalized with 100 percent consensus. 

The baseline questions comprised items on demographic details and self-assessment of training needs, which were rated on a four-point scale labeled from ‘highly relevant’, ‘rather relevant’, ‘rather irrelevant’ and ‘irrelevant’. In four sections of the questionnaire, items on training-specific needs were categorized into “teaching and learning theory”, “presentation and communication”, “examination” and “mentoring”. The items were based on learning objectives focusing on the improvement of teaching in the German state of Bavaria [32]. They were built upon using the practical experience of the members of the German Association for Psychiatry, Psychotherapy and Psychosomatics, having in mind a general approach to teaching medical doctors rather than specialists (psychiatry). Therefore, the learning goals were not limited to psychiatric teaching but were in general relevant for other teaching disciplines.

All questions were scaled according to a learning taxonomy and rated on a four-point scale labeled from least important on the left to most important on the right: “not relevant for me”, “just want to learn the facts”, “want to train and get feedback”, and “want to be able to teach others”. In another section, the participants were asked about their preferences for online or in-person teaching.

The questionnaire was available online on SoSci Survey, and the link was distributed via email to medical faculty members in German-speaking countries. The survey was conducted from May 2021 until May 2022 (reminder emails were sent out in May 2021, June 2021 and April 2022).

### Data analysis

Statistical analysis was carried out using SPSS v.28 (IBM, Armonk, NY, USA). Figures were created with Prism v.6 (GraphPad Software Inc., La Jolla, CA, USA). Data are reported in relative frequencies and as the modal with interquartile range (IQR). Differences between groups were calculated with the Mann-Whitney U test. Correlations were calculated with the Spearman correlation coefficient. The results were considered significant at p<0.05, while p<0.1 was considered a trend.

## Results

Overall, 97 respondents (male=55, female=42; mean age=40.6 years) from 19 medical schools in Germany and Switzerland completed the questionnaire. Of the 97 respondents, 63 were from the originally contacted university clinics while 34 were from teaching hospitals and other clinics reached via snowballing. The sample characteristics are summarized in table 1 (Tab. 1).

### Didactic competence

Among the 97 respondents, 97.9% considered didactic competence (DC) relevant for teaching medical students, with 70.1% finding it to be highly relevant. DC was rated as significantly more relevant for teaching medical students than for career trajectory (p<0.0001) or informing patients (p<0.05). DC for training colleagues was also viewed as more relevant than for career trajectory (p<0.05).

**Figure 1:**
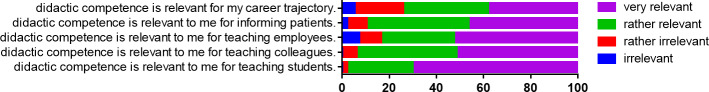
The perceived importance for didactic competence as rated by the participants

Descriptive values are provided in figure 1 (Fig. 1).

Specialist physicians (SP) deemed DC more relevant for teaching colleagues (p<0.0001), employees (p<0.0001) and students (p=0.046) than residents. When grouped according to their professional goals, aspiring professors deemed DC more relevant for career trajectory (p=0.007) than those who did not aim to become professors. Respondents aiming for tenure track differed from the rest of respondents in their interest regarding training on how to give feedback (p=0.021) and perform evaluations (p=0.017). Furthermore, they viewed DC as significantly more relevant for their career trajectory (p=0.026). Overall, respondents aspiring to work in private practice thought of DC as less relevant for training their employees (p=0.006).

Work experience in years was positively correlated with interest in examination didactics (ρ=0.200, p=0.05) and negatively correlated with interest in simulated patients (ρ=-0.251, p=0.013) and roleplay (ρ=-0.223, p=0.028).

### Desired training topics

**Figure 2:**
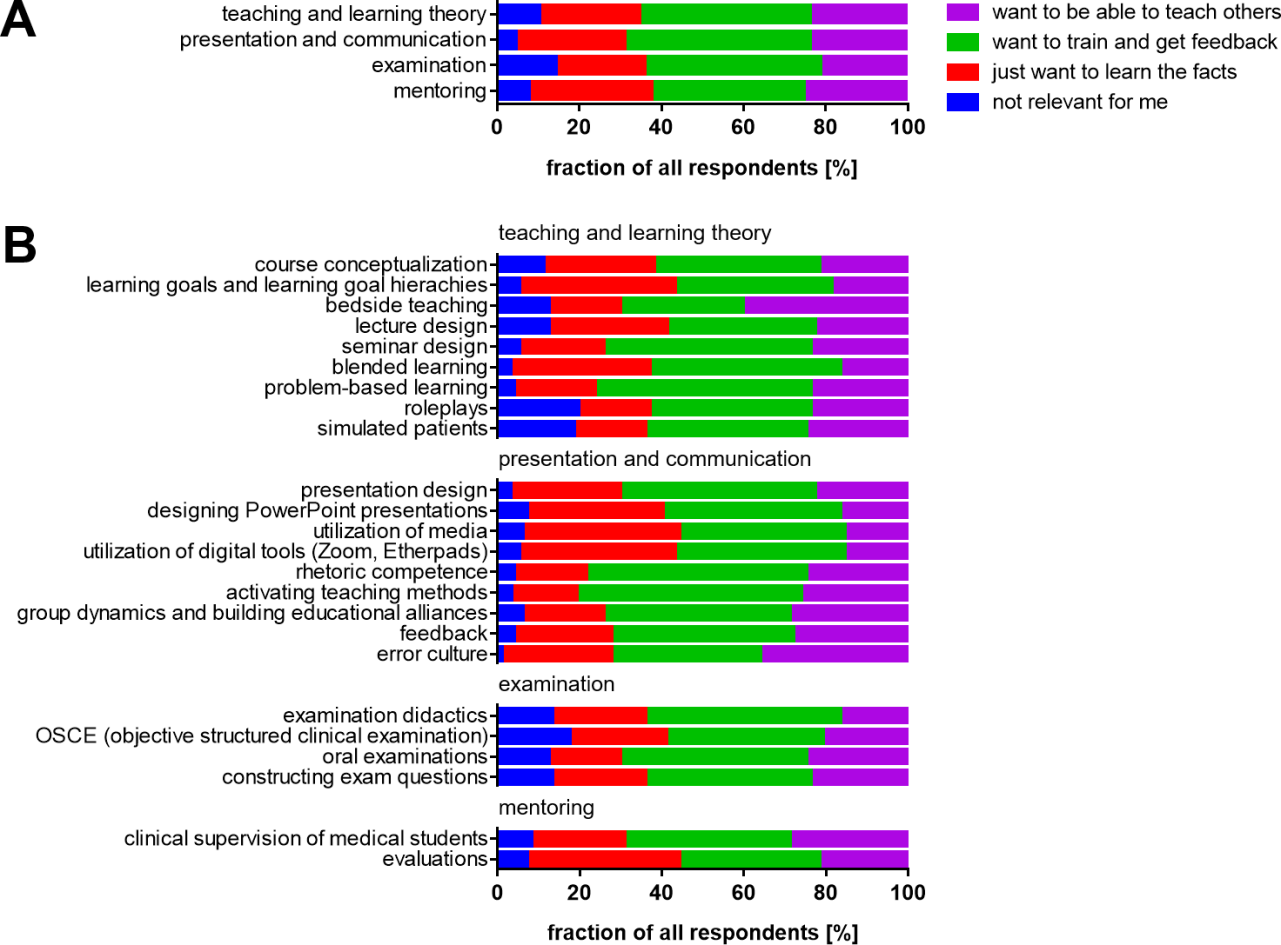
All learning goals as rated for relevancy by the participants

Questions on training needs were subsumed into categories of “teaching and learning theory”, “presentation and communication”, “examination” and “mentoring”. A majority of respondents (68.9%) expressed interest in topics regarding “presentation and communication” (see figure 2 A (Fig. 2)).

The participants rated “bedside teaching” and “error culture” as the most relevant training topics, with 70.1% and 72.2%, respectively, wanting to learn how to train others or to practice these subjects themselves. Learning about “clinical supervision of medical students” was deemed relevant by 69.1% for wanting to learn how to train others or to practice these subjects themselves. Learning about “group dynamics and building educational alliances” was deemed relevant by 74.2% for wanting to learn how to train others or to practice these subjects themselves. Learning about “giving feedback” was deemed relevant by 72.2% of respondents for wanting to learn how to train others or to practice these subjects themselves. For “activating teaching methods” and “rhetoric”, 81.3% and 78.4% of respondents respectively, wished to learn how to train others or to practice these subjects themselves, followed by “problem-based learning”, chosen by 76.3% of respondents.

Respondents wanting to become senior physicians displayed significantly higher interest than others in the supervision of medical students in the practical year (p=0.007) and bedside teaching (p=0.017). Participants were least interested in learning how to set goals and perform evaluations, with 38.1% of respondents wanting to learn only facts about “learning goal hierarchies and formulating learning goals”, and 37.1% expressing a need for learning only facts about “evaluations” (see figure 2 B (Fig. 2)).

### Group comparisons

Residents were more interested in “bedside teaching” (p=0.004) and “roleplay” (p=0.047) than SPs. They also showed higher interest in “bedside teaching” (p=0.003) than psychologists. Residents expressed deeper interest in “designing PowerPoint presentations” (p=0.032) and “clinical supervision of medical students” (p=0.027) than psychologists.

**Figure 3:**
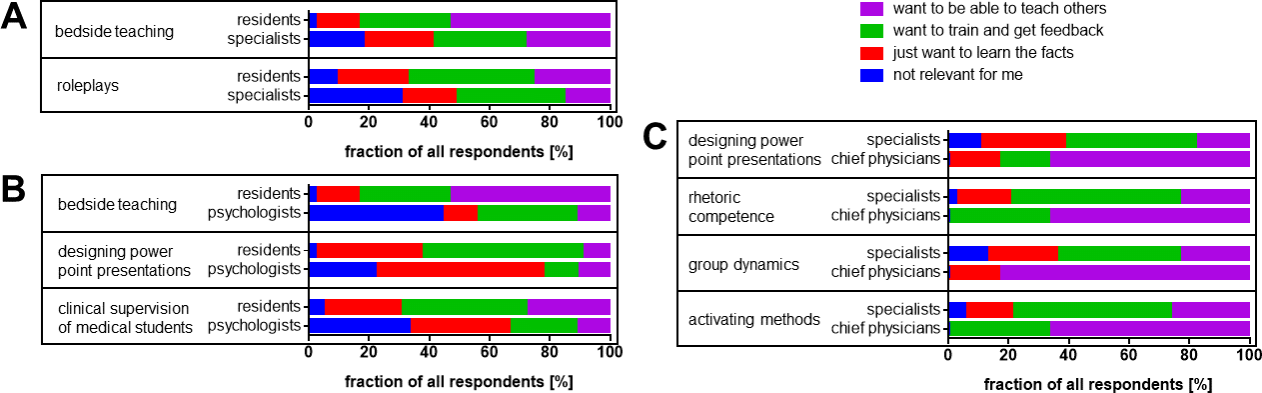
Differences in learning goals when compared among different professional groups

Chief physicians (CPs) were more interested in “group dynamics and building educational alliances” (p=0.023), “rhetoric competence” (p=0.030), “designing PowerPoint presentations” (p=0.041) and “activating teaching methods” (p=0.044) compared to SPs. Figure 3 (Fig. 3) illustrates these findings.

The strongest differences between those wanting to become professors (n=11) and those who did not (n=86) were observed in the subjects of formulating learning goals (p<0.001), blended learning (p<0.001) and evaluations (p=0.001). Respondents aspiring to work in private practices were significantly more interested in bedside teaching (p=0.002).

Almost all survey items regarding topics of medical didactics were significantly positively correlated with each other. The only exceptions were interest in “designing PowerPoint presentations” and “inclusion of media” showing only a trend for a positive correlation with an interest in “simulated patients” and no correlation at all with an interest in “roleplay”.

### Preferences for didactic workshops

**Figure 4:**
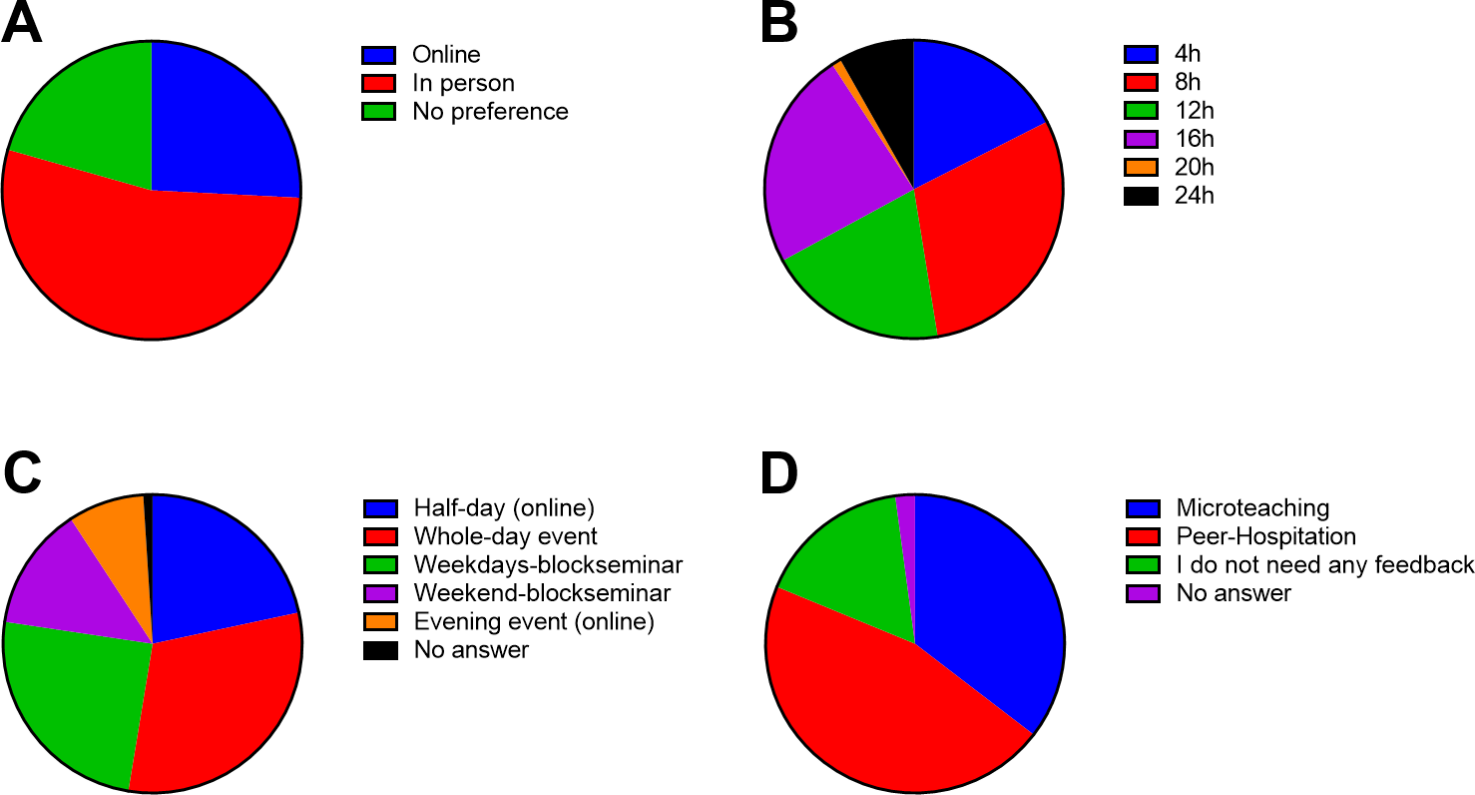
An overview of the preferences for didactic workshops from our respondents

Most respondents preferred in-person teaching over online courses, with 25.8% preferring online teaching (see figure 4 (Fig. 4)). In addition, they voted for a time frame between 8 hours and 16 hours. Most respondents preferred whole-day workshops and block seminars during the week. Additionally, they wanted to be given feedback using a peer-shadowing format or microteaching.

## Discussion

This survey aimed to explore the need for didactic training perceived by physicians involved in teaching at psychiatry departments of medical schools in three German-speaking regions. Using a purpose-derived questionnaire, we asked which teaching skills and methods were the most relevant for medical educators in psychiatry and compared them to identify the differences in preferences among respondent groups. While the majority of physicians rated didactic training relevant for gaining necessary skills for teaching medical students, there were significant differences between educational preferences and the desired teaching topics of professionals at different stages of their careers. Learning how to deliver bedside teaching and how to implement a sound error culture were rated as the most important topics. Our findings highlight the need for designing targeted educational interventions for medical educators at different stages of their careers. These results can be useful to design purpose-derived workshops according to the needs of the intended participants, in consideration with training standards, subject-didactic recommendations and curriculum designs.

### Didactic competence

An overwhelming majority of our respondents considered didactic competence relevant for teaching medical students, with over two-thirds deeming it “highly relevant”. Teaching is a core clinical skill that requires training and practice accompanied by constructive feedback. Although physicians are expected to teach at medical schools even as residents, they mostly do not receive any formal training in teaching as students during undergraduate medical education. Our findings showed that physicians and psychologists recognized the importance of training in teaching methods and considered it to be more relevant for teaching medical students than for other professional aspects. Interestingly, teaching medical students took precedence over all other aspects of informing patients, training fellow colleagues or employees and especially over career aspirations. This points towards a desire to obtain more training to improve their didactic skills. These results are in line with previous studies that have also noted the absence of opportunities for physicians to obtain educational training [33], [34].

Additionally, work experience was positively correlated with interest in examination didactics, suggesting that medical didactics for teaching purposes were valued more by those with longer professional experience. This may be explained by the fact that more experienced professionals are regularly involved in conducting exams than their junior colleagues, and therefore give higher importance to examination didactics. Previous studies have shown that medical educators at different stages of their careers have different interests and priorities [35], [36].

### Desired training topics

When asked which topics they would like to learn at didactic workshops, the participants rated “bedside teaching” and “error culture” as the most important learning goals. All medical treatments are inherently risky, and medical practitioners encounter patient safety issues every day [37]. Although managing errors in medicine is an important aspect for patients as well as caregivers, progress on adding patient safety topics to the core medical curriculum in Germany has been slow [38]. The NKLM 1.0 [12], which was published in 2015, described 13 learning objectives related to patient safety. In recent years, error culture has gained importance as a teaching topic in the German medical education guidelines, as patient representative associations have advocated for a better focus on error management. The NKLM 2.0 released in 2021 now describes 54 learning objectives related to patient safety [39]. Workshops on patient safety training to develop a positive error culture may be a useful stopgap solution until the medical curricula are modified to include more teaching on this topic.

Bedside teaching is an established tool for enhancing medical students’ learning experience and skills [40], [41]. Our results show that the majority of physicians are interested in being trained in bedside teaching at workshops, even though the use of bedside teaching has significantly declined in recent decades because of rapid improvements in biomedical technologies, simulation techniques and high patient turnover at hospitals [42]. Junior doctors have increasingly taken up this role of clinical teaching, and medical students appear to be more comfortable being taught by junior doctors who they consider more approachable and less intimidating [40]. In our results, residents were more interested in bedside teaching and roleplay, while chief physicians were more interested in faculty development. These findings reiterate that training workshops should be designed to accommodate and support teaching physicians according to the specific needs of the participants. Additionally, different teaching methods should be utilized for different professional groups, such as using simulated patients for the training of resident physicians and supervisory training for senior physicians.

Training in designing seminars, communication skills and activating teaching methods was also rated to be important, while basic concepts such as training on learning goals and evaluations were regarded as less relevant overall.

Using the data on desired training topics, we thematically categorized the training needs into four overarching themes: “teaching and learning concepts”, “presentation and communication”, “examinations” and “mentoring”. Over two-thirds of our respondents rated “communication and presentation” as the most desired aspect of teachers’ training. A physician profile typically consists of clinical, research and educational/academic-oriented tasks. While some individuals might have all three profiles to a certain extent, those interested in an academic career are most likely to expand their didactic skills, whereby communication and presentations play a vital role. Presentations are also important tools for staff involved in teaching as well as clinical research.

Teaching skills have been encouraged as a professional competence for doctors, and several countries have adopted national guidelines to set learning goals for future physicians [10], [12]. The Heidelberg Medical School introduced an integrated qualification program for student tutors in 2010 [43], whereby students are prepared for teaching activities through training in didactics, group management, Problem-based learning (PBL) and physical examination techniques as well as on planning and performance of courses and giving feedback on their activities. Previous studies have recommended continuing such training modules throughout postgraduate education as well as into practice [18], since teaching principles, skills and techniques are an evolving process. While programs to train medical students in teaching are fairly common these days [7], [44], practicing physicians mainly have to pursue master’s or doctoral programs or utilize other faculty development offers or fellowships [45], [46], [47], [48]. Therefore, teachers’ training workshops for physicians can go a long way to fill this conspicuous gap in clinician-educator competencies.

### Preferences for didactic workshops

Most participants in our study preferred in-person teaching over online courses, with only one-fourth of them preferring online teaching. In addition, they mostly voted for a time frame between 8 hours and 16 hours and preferred whole-day workshops and block seminars during the week. While a regular online workshop in the evening was less preferable, it could be much easier to organize logistically for professional medical educators. In the past two years, teaching activities at medical schools have undergone a significant shift from ward training to online teaching owing to pandemic-related restrictions [49]. Since our survey was conducted during this time period, the preference for in-person rather than online workshops might have been biased by the “pandemic fatigue” caused by online-oriented activities. While ward training and bedside teaching are cornerstones of medical education, teachers should be trained using diverse modalities. Online as well as blended learning formats have been shown to produce similar or even better results in medical education [50], [51], [52]. When asked about their preferences for receiving feedback on their teaching, most respondents were interested in receiving feedback via peer shadowing or microteaching, both of which are seldom used in didactic workshops. 

### Limitations

Our survey was distributed at 54 medical faculties across Germany, Austria and Switzerland. However, the sample size could be deemed too small to be able to derive generalizable results since we received responses from 19 schools contacted and some others via snowball sampling. The low response rate could be explained via several factors, but the COVID-19 pandemic played a major role. Healthcare professionals were extremely overburdened during the past two years and medical educators were coping with the enormous task of transforming in-person training to online teaching protocols. Therefore, teaching staff would not have prioritized participating in research activities during the pandemic.

While most of our respondents deemed teachers’ training highly relevant for gaining necessary skills for teaching medical students, these findings should be interpreted cautiously since our sample mainly consisted of physicians and psychologists already teaching at medical faculties. It may be assumed that physicians and psychologists with a preference for medical education were more likely to respond to the survey than those who were not interested in didactics. This selection bias could have played a decisive role in the choice to prioritize the education of students over their career aspirations.. 

Additionally, physicians who might participate in a medical didactics workshop are already more interested in didactics than those who are not involved in teaching. A larger sample size without any restrictions for specialist faculty members could be repeated to obtain further results.

## Conclusions

In this survey, we explored the didactic training needs of physicians teaching at medical schools. Our results showed that physicians considered didactic competence highly relevant for teaching medical students and that they could benefit from training workshops designed to improve their teaching skills. However, there were significant differences between the training needs and preferences of professionals at different stages of their careers. Most physicians, at some point in their career, are expected to take up responsibilities for teaching their colleagues and medical students. Some medical schools have initiated training courses for medical education for future physicians, but most students are expected to assume teaching responsibilities without formal training. In the absence of such courses, didactic workshops can provide the necessary skills and experience for the development, implementation and evaluation of medical educators. Our findings highlight the need for designing targeted educational interventions for medical educators at different stages of their careers. Our results can be useful in designing purpose-derived workshops according to the needs of the intended participants. Workshops could be tailored according to the characteristics of the subgroups based on the findings relevant to each subgroup, such as training in bedside teaching for residents and training on how to conduct examinations for chief physicians. Furthermore, individualized workshops might be the most beneficial. Future research should identify the prevalence and content of courses on teacher training for postgraduate medical students in Germany and/or German-speaking countries.

## Notes

### Author contributions

The authors Gian-Marco Kerstenx and Philipp Spitzer have contributed equally and share the last authorship.

- Project administration: PS, JU, CR, GMK
- Conceptualization: PS, JU, CR, GMK, KK, TF, JS-K, SP, GMB, DJ, SS, DR-E, IK, MA, CR, JU
- Methodology: PS, JU, CR, GMK, KK, TF, JS-K, SP, GMB, DJ, SS, DR-E, IK, MA, CR, JU
- Formal analysis: GMK, FB, PS
- Writing – original draft: AZ, FB, GMK, PS
- Review and editing: KK, TF, JS-K, SP, GMB, DJ, SS, DR-E, IK, MA, CR, JU

All authors have read and agreed to the final version of the manuscript.

### Authors’ ORCIDs

- Franziska Baessler: [0000-0001-7280-9675]
- Ali Zafar: [0000-0002-1305-7407]
- Katja Koelkebeck: [0000-0002-0469-3997]
- Thomas Frodl: [0000-0002-8113-6959]
- Jörg Signerski-Krieger: [0000-0001-7873-4133]
- Severin Pinilla: [0000-0002-0797-2043]
- Deborah Janowitz: [0000-0003-4324-9298]
- Sven Speerforck: [0000-0002-9281-8461]
- Daniela Roesch-Ely: [0000-0001-6877-2646]
- Ina Kluge: [0009-0009-7400-1368]
- Janine Utz: [0009-0002-7227-3844]
- Philipp Spitzer: [0000-0001-9555-605X]

### Ethical approval

In the German state of Bavaria, ethics approval was not mandatory for studies using anonymous data collection methods according to Section 15 (Research) of the Professional Code of Conduct for Physicians in Bavaria.

### Data availability

Anonymized datasets generated and analyzed for this study can be made available by the corresponding author on reasonable request.

## Acknowledgements

We are thankful to the members of the German Society for Psychiatry and Psychotherapy, Psychosomatics and Neurology (DGPPN) for their help in conceptualizing the questionnaire. Franziska Baessler is supported under the Olympia-Morata fellowship program of the Heidelberg University Medical Faculty.

## Competing interests

The authors declare that they have no competing interests. 

## Supplementary Material

Umfrage DGPPN Medizindidaktik - in German

## Figures and Tables

**Table 1:**
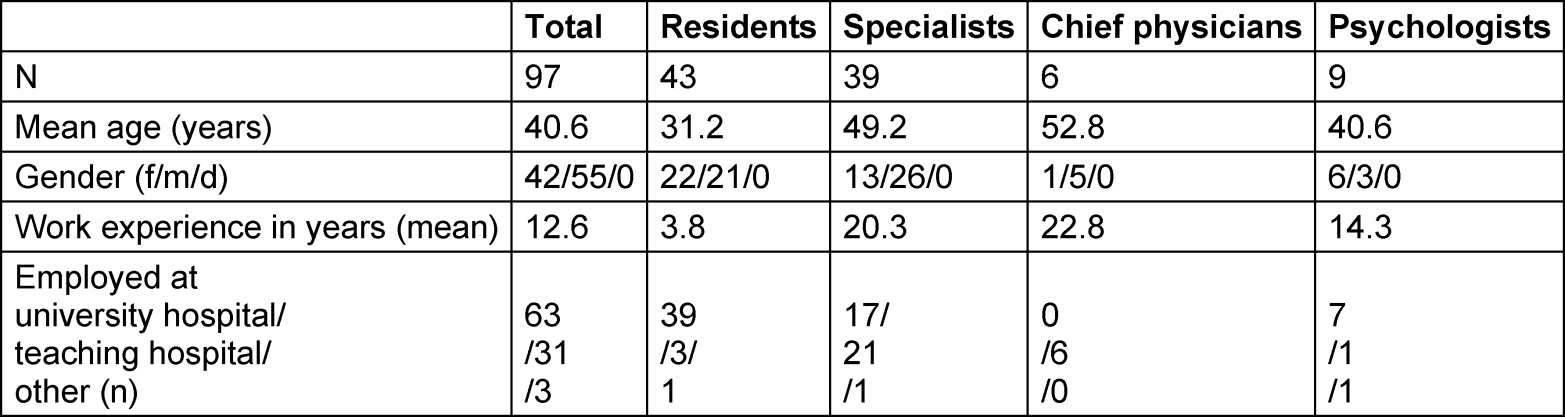
Sociodemographic characteristics of the sample
