# Supplementary material for: What do the teachers want? A targeted needs assessment survey for prospective didactic training of psychiatry medical educators
Source: GMS J Med Educ. 2024 Apr 15;41(2):Doc18. doi: 10.3205/zma001673 (PMC11106569; doi:10.3205/zma001673)
Supplement: Umfrage DGPPN Medizindidaktik - in German [file JME-41-18-s-001.pdf]

Attachment 1: Umfrage DGPPN Medizindidaktik – in German

Seite 1 Einführung

Liebe Kolleg*innen,

vielen Dank, dass Sie sich die Zeit nehmen, unseren Fragebogen zu beantworten.
Vermutlich gehört auch bei Ihnen die studentische Lehre zum Tagesgeschäft. Wir, vom Referat Medizindidaktik der DGPPN, planen einen kostenfreien und für alle Ärzt*innen und Psycholog*innen an Lehrkrankenhäusern zugänglichen Workshop zur Vermittlung von didaktischen Grundlagen. Um diesen Workshop auf Ihre Bedürfnisse anzupassen, würden wir Sie bitten, die nachfolgenden Fragen zu einem derartigen Workshop zu beantworten. Die Dauer dafür beträgt 5 bis 10 Minuten. Die Datenerhebung erfolgt streng anonymisiert und dient ausschließlich dem Zweck der Workshop-Planung, sowie einer wissenschaftlichen Auswertung.

Viele Grüße
Philipp Spitzer
im Auftrag des Referats Medizindidaktik (DGPPN)

Seite 2 Alter und Geschlecht
Alter:

Geschlecht:

m	w	d

Seite 3 Arbeit und Karriere

Ich arbeite aktuell in:

**Deutschland**	**Schweiz**	**Österreich**	**andere – und zwar**

Mein Arbeitsplatz ist ein/eine:

**Universitätsklinik**	**Akademisches Lehrkrankenhaus**	**Lehrpraxis**	**andere – und zwar**

Mein Arbeitsplatz gehört zur Universität:

**RWTH Aachen**	**Universität Augsburg**	**Humboldt-Universität zu Berlin**	**Ruhr-Universität Bochum**	**Friedrich-Wilhelms-Universität Bonn**	**Technische Universität Dresden**
**Heinrich-Heine-Universität Düsseldorf**	**Friedrich-Alexander-Universität Erlangen-Nürnberg**	**Universität Duisburg-Essen**	**Johann-Wolfgang-Goethe-Universität Frankfurt am Main**	**Albert-Ludwigs-Universität Freiburg**	**Justus-Liebig-Universität Gießen**
**Georg-August-Universität Göttingen**	**Ernst-Moritz-Arndt-Universität Greifswald**	**Martin-Luther-Universität Halle-Wittenberg**	**Universität Hamburg**	**Medizinische Hochschule Hannover**	**Ruprecht-Karls-Universität Heidelberg**
**Universität des Saarlandes Homburg**	**Friedrich-Schiller-Universität Jena**	**Christian-Albrechts-Universität Kiel**	**Universität Köln**	**Universität Leipzig**	**Universität Lübeck**

Anhang 1 zu Baessler F, Zafar A, Koelkebeck K, Frodl T, Signerski-Krieger J, Pinilla S, Barth GM, Janowitz D, Speerforck S, Roesch-Ely D, Kluge I, Aust M, Rauch C, Utz J, Kersten GM, Spitzer P. *What do the teachers want? A targeted needs assessment survey for prospective didactic training of psychiatry medical educators.* GMS J Med Educ. 2024;41(2):Doc18. DOI: 10.3205/zma001673

**Otto-von-Guericke-Universität Magdeburg**	**Medizinische Fakultät Mannheim der Universität Heidelberg**	**Johannes-Gutenberg-Universität Mainz**	**Philipps-Universität Marburg**	**Ludwig-Maximilians-Universität München**	**Technische Universität München**
**Westfälische Wilhelms-Universität Münster**	**Carl-von-Ossietzky-Universität Oldenburg**	**Universität Regensburg**	**Universität Rostock**	**Eberhard-Karls-Universität Tübingen**	**Universität Ulm**
**Julius-Maximilians-Universität Würzburg**	**Universität Basel**	**Universität Bern**	**Universität Fribourg**	**Universität Genève**	**Universität Lausanne**
**Universität Neuchâtel**	**Universität Zürich**	**Universität Wien**	**Universität Innsbruck**	**Universität Graz**	**Johannes Kepler Universität Linz**
**Paracelsus Medizinische Privatuni-versität in Salzburg**	**Paracelsus Medizinische Privatuni-versität in Nürnberg**	**Danube Private University in Krems**	**Karl Landsteiner Privatuniversität für Gesundheitswissen-schaften**	**Sigmund Freud Privatuni-versität**	**andere – und zwar**

Ich arbeite als:

**Ärzt*in in der fachärztlichen Weiterbildung**	**Fachärzt*in**	**Psycholog*in**	**Anderes – und zwar**

Ich arbeite seit ______ **Jahren.**

Mein berufliches Ziel:

**Fachärzt*in in der Niederlassung**	**Fachärzt*in am Klinikum**	**Oberärzt*in**
**Chefärzt*in**	**Promotion**	**Habilitation**
**Professur**	**Andere – und zwar**	**keine Angabe**

Seite 4 Lehr- und Lernkonzepte

Diese Themen möchte ich im Seminar bearbeiten in Sachen „Lehr- und Lernkonzepte“

**Konzeptualisierung einer Lernveranstaltung**	**Das ist für mich nicht relevant**	**Dazu möchte ich nur Fakten lernen**	**Das möchte ich trainieren und Feedback erhalten**	**Dazu möchte ich andere unterrichten können**
**Lernzielhierarchien und Formulieren von Lernzielen**	**Das ist für mich nicht relevant**	**Dazu möchte ich nur Fakten lernen**	**Das möchte ich trainieren und Feedback erhalten**	**Dazu möchte ich andere unterrichten können**

**Bedside teaching**	**Das ist für mich nicht relevant**	**Dazu möchte ich nur Fakten lernen**	**Das möchte ich trainieren und Feedback erhalten**	**Dazu möchte ich andere unterrichten können**
**Vorlesungen gestalten**	**Das ist für mich nicht relevant**	**Dazu möchte ich nur Fakten lernen**	**Das möchte ich trainieren und Feedback erhalten**	**Dazu möchte ich andere unterrichten können**
**Seminare gestalten**	**Das ist für mich nicht relevant**	**Dazu möchte ich nur Fakten lernen**	**Das möchte ich trainieren und Feedback erhalten**	**Dazu möchte ich andere unterrichten können**
**Integriertes Lernen/blended learning (Eine Lernform bei der die Vorteile von Präsenzveranstaltungen und E-Learning kombiniert werden)"**	**Das ist für mich nicht relevant**	**Dazu möchte ich nur Fakten lernen**	**Das möchte ich trainieren und Feedback erhalten**	**Dazu möchte ich andere unterrichten können**
**Problembasiertes/Problemorientiertes Lernen (Eine Lernform, deren Charakteristikum es ist, dass die Lernenden weitgehend selbständig eine Lösung für ein vorgegebenes Problem finden sollen)"**	**Das ist für mich nicht relevant**	**Dazu möchte ich nur Fakten lernen**	**Das möchte ich trainieren und Feedback erhalten**	**Dazu möchte ich andere unterrichten können**
**Rollenspiele**	**Das ist für mich nicht relevant**	**Dazu möchte ich nur Fakten lernen**	**Das möchte ich trainieren und Feedback erhalten**	**Dazu möchte ich andere unterrichten können**
**SimulationspatientInnen**	**Das ist für mich nicht relevant**	**Dazu möchte ich nur Fakten lernen**	**Das möchte ich trainieren und Feedback erhalten**	**Dazu möchte ich andere unterrichten können**

Seite 5 Präsentation und Kommunikation

Diese Themen möchte ich im Seminar bearbeiten in Sachen „Präsentation und Kommunikation“

**Vortragsgestaltung**	**Das ist für mich nicht relevant**	**Dazu möchte ich nur Fakten lernen**	**Das möchte ich trainieren und Feedback erhalten**	**Dazu möchte ich andere unterrichten können**
**Gestaltung von Powerpointfolien**	**Das ist für mich nicht relevant**	**Dazu möchte ich nur Fakten lernen**	**Das möchte ich trainieren und Feedback erhalten**	**Dazu möchte ich andere unterrichten können**

**Einbindung von Medien**	**Das ist für mich nicht relevant**	**Dazu möchte ich nur Fakten lernen**	**Das möchte ich trainieren und Feedback erhalten**	**Dazu möchte ich andere unterrichten können**
**Umgang mit digitalen Hilfsmitteln (Zoom, Etherpads)**	**Das ist für mich nicht relevant**	**Dazu möchte ich nur Fakten lernen**	**Das möchte ich trainieren und Feedback erhalten**	**Dazu möchte ich andere unterrichten können**
**Rhetorik**	**Das ist für mich nicht relevant**	**Dazu möchte ich nur Fakten lernen**	**Das möchte ich trainieren und Feedback erhalten**	**Dazu möchte ich andere unterrichten können**
**aktivierende Lehre**	**Das ist für mich nicht relevant**	**Dazu möchte ich nur Fakten lernen**	**Das möchte ich trainieren und Feedback erhalten**	**Dazu möchte ich andere unterrichten können**
**Beziehungsgestaltung und Gruppendynamik**	**Das ist für mich nicht relevant**	**Dazu möchte ich nur Fakten lernen**	**Das möchte ich trainieren und Feedback erhalten**	**Dazu möchte ich andere unterrichten können**
**Feedback**	**Das ist für mich nicht relevant**	**Dazu möchte ich nur Fakten lernen**	**Das möchte ich trainieren und Feedback erhalten**	**Dazu möchte ich andere unterrichten können**
**Fehlerkultur**	**Das ist für mich nicht relevant**	**Dazu möchte ich nur Fakten lernen**	**Das möchte ich trainieren und Feedback erhalten**	**Dazu möchte ich andere unterrichten können**

Seite 6 Prüfen + Entwickeln und Begleiten

Diese Themen möchte ich im Seminar bearbeiten in Sachen „Prüfen“

**Prüfungsdidaktik**	**Das ist für mich nicht relevant**	**Dazu möchte ich nur Fakten lernen**	**Das möchte ich trainieren und Feedback erhalten**	**Dazu möchte ich andere unterrichten können**
**OSCE (objective structured clinical examination)**	**Das ist für mich nicht relevant**	**Dazu möchte ich nur Fakten lernen**	**Das möchte ich trainieren und Feedback erhalten**	**Dazu möchte ich andere unterrichten können**
**Mündliche Prüfungen**	**Das ist für mich nicht relevant**	**Dazu möchte ich nur Fakten lernen**	**Das möchte ich trainieren und Feedback erhalten**	**Dazu möchte ich andere unterrichten können**
**Stellen von Prüfungsfragen**	**Das ist für mich nicht relevant**	**Dazu möchte ich nur Fakten lernen**	**Das möchte ich trainieren und Feedback erhalten**	**Dazu möchte ich andere unterrichten können**

Diese Themen möchte ich im Seminar bearbeiten in Sachen „Entwickeln und Begleiten“

**Betreuung von PJlerInnen (Wahljahrstudierende/UnterassistentInnen) und FamulantInnen**	**Das ist für mich nicht relevant**	**Dazu möchte ich nur Fakten lernen**	**Das möchte ich trainieren und Feedback erhalten**	**Dazu möchte ich andere unterrichten können**
**Evaluationen**	**Das ist für mich nicht relevant**	**Dazu möchte ich nur Fakten lernen**	**Das möchte ich trainieren und Feedback erhalten**	**Dazu möchte ich andere unterrichten können**

Seite 7 Freitext

Diese Themen würden mich zusätzlich interessieren

Seite 8 Rahmenbedingungen

Online vs Präsenz

**Ich würde lieber an einem Seminar für Medizindidaktik teilnehmen, dass…**	**… Online stattfindet.**	**… in Präsenz stattfindet.**	**Keine Präferenz**

Zeitlicher Rahmen

**Als gesamten zeitlichen Umfang würde ich mir wünschen**	**4h**	**8h**	**12h**	**16h**	**20h**	**24h**

Rahmen

**Zur Aufteilung der Seminar-stunden würde ich mir folgendes Format wünschen**	**halbtägige Veranstaltung (nur online)**	**ganztägige Veranstaltung**	**Blockveranstaltung unter der Woche**	**Blockveranstaltung am Wochenende**	**abendliche Veranstaltung (nur online)**

Feedback

**Ich würde mir ein Feedback zur eigenen Lehre wünschen**	**Microteaching (videogestütztes Lehrtraining mit Feedback)**	**Organisation einer Peer-Hospitation (gegenseitiger Besuch von Lehrveranstaltungen mit anschließendem Feedback)**	**anderes – und zwar**	**Ich brauche kein Feedback**

Seite 9 Persönliche Einschätzung

Persönliche Einschätzung

**Didaktische Kompetenz ist für mich für die studentische Lehre relevant.**	**sehr relevant**	**eher ja**	**eher nein**	**nicht relevant**	**nicht beantwortet**
**Didaktische Kompetenz ist für mich relevant, um KollegInnen Wissen zu vermitteln.**	**sehr relevant**	**eher ja**	**eher nein**	**nicht relevant**	**nicht beantwortet**
**Didaktische Kompetenz ist für mich relevant, um meine Mitarbeiter*innen weiterzubilden.**	**sehr relevant**	**eher ja**	**eher nein**	**nicht relevant**	**nicht beantwortet**
**Didaktische Kompetenz ist für mich relevant, um meine Patient*innen zu informieren.**	**sehr relevant**	**eher ja**	**eher nein**	**nicht relevant**	**nicht beantwortet**
**Didaktische Kompetenz ist für meine Karriere relevant.**	**sehr relevant**	**eher ja**	**eher nein**	**nicht relevant**	**nicht beantwortet**

Seite 10 Freies Feedback

Haben Sie weitere Wünsche und Anregungen zu unserem Workshop, dann teilen Sie uns diese bitte hier mit:

Seite 11 Letzte Seite

Vielen Dank für Ihre Teilnahme!

Wir möchten uns für Ihre Mithilfe bedanken.

Ihre Antworten wurden gespeichert, Sie können das Browser-Fenster nun schließen.

Philipp Spitzer, im Auftrag des Referats Medizindidaktik (DGPPN)

Anhang 1 zu Baessler F, Zafar A, Koelkebeck K, Frodl T, Signerski-Krieger J, Pinilla S, Barth GM, Janowitz D, Speerforck S, Roesch-Ely D, Kluge I, Aust M, Rauch C, Utz J, Kersten GM, Spitzer P. *What do the teachers want? A targeted needs assessment survey for prospective didactic training of psychiatry medical educators.* GMS J Med Educ. 2024;41(2):Doc18. DOI: 10.3205/zma001673
